# Creating and analyzing pathway and protein interaction compendia for modelling signal transduction networks

**DOI:** 10.1186/1752-0509-6-29

**Published:** 2012-05-01

**Authors:** Daniel C Kirouac, Julio Saez-Rodriguez, Jennifer Swantek, John M Burke, Douglas A Lauffenburger, Peter K Sorger

**Affiliations:** 1 Department of Biological Engineering, Massachusetts Institute of Technology, Cambridge, MA, 02139, USA; 2 European Bioinformatics Institute (EMBL-EBI), Wellcome Trust Genome Campus, Cambridge, UK, CB10 1SD; 3 Immunology & Inflammation, Boehringer Ingelheim Pharmaceuticals, Ridgefield, CT, 06877-0368, USA; 4 Department of Systems Biology, Harvard Medical School, Boston, MA, 02115, USA; 5 Merrimack Pharmaceuticals, Cambridge, MA, 02139, USA

## Abstract

**Background:**

Understanding the information-processing capabilities of signal transduction networks, how those networks are disrupted in disease, and rationally designing therapies to manipulate diseased states require systematic and accurate reconstruction of network topology. Data on networks central to human physiology, such as the inflammatory signalling networks analyzed here, are found in a multiplicity of on-line resources of pathway and interactome databases (Cancer CellMap, GeneGo, KEGG, NCI-Pathway Interactome Database (NCI-PID), PANTHER, Reactome, I2D, and STRING). We sought to determine whether these databases contain overlapping information and whether they can be used to construct high reliability prior knowledge networks for subsequent modeling of experimental data.

**Results:**

We have assembled an ensemble network from multiple on-line sources representing a significant portion of all machine-readable and reconcilable human knowledge on proteins and protein interactions involved in inflammation. This ensemble network has many features expected of complex signalling networks assembled from high-throughput data: a power law distribution of both node degree and edge annotations, and topological features of a “bow tie” architecture in which diverse pathways converge on a highly conserved set of enzymatic cascades focused around PI3K/AKT, MAPK/ERK, JAK/STAT, NFκB, and apoptotic signaling. Individual pathways exhibit “fuzzy” modularity that is statistically significant but still involving a majority of “cross-talk” interactions. However, we find that the most widely used pathway databases are highly inconsistent with respect to the actual constituents and interactions in this network. Using a set of growth factor signalling networks as examples (epidermal growth factor, transforming growth factor-beta, tumor necrosis factor, and wingless), we find a multiplicity of network topologies in which receptors couple to downstream components through myriad alternate paths. Many of these paths are inconsistent with well-established mechanistic features of signalling networks, such as a requirement for a transmembrane receptor in sensing extracellular ligands.

**Conclusions:**

Wide inconsistencies among interaction databases, pathway annotations, and the numbers and identities of nodes associated with a given pathway pose a major challenge for deriving causal and mechanistic insight from network graphs. We speculate that these inconsistencies are at least partially attributable to cell, and context-specificity of cellular signal transduction, which is largely unaccounted for in available databases, but the absence of standardized vocabularies is an additional confounding factor. As a result of discrepant annotations, it is very difficult to identify biologically meaningful pathways from interactome networks *a priori*. However, by incorporating prior knowledge, it is possible to successively build out network complexity with high confidence from a simple linear signal transduction scaffold. Such reduced complexity networks appear suitable for use in mechanistic models while being richer and better justified than the simple linear pathways usually depicted in diagrams of signal transduction.

## Background

Cells monitor their external environment, transmit information across membranes, and make cell fate decisions using multi-protein receptor-mediated signal transduction networks [1] that represent the “perceptual” circuits of a cell [2]. Many diseases are now understood to result from disruption of cellular signal transduction cascades and the proliferative, metabolic and differentiation programs they control [3]. Signal transduction has traditionally been represented as a series of discrete enzymatic cascades, a simplification that is useful when the goal is to understand the activities of individual proteins and protein complexes. However, it is increasingly apparent that linear representations are insufficient, and that “canonical” signal transduction cascades are components of an interconnected web of molecular circuitry that includes extensive cross talk among different receptors [4-7]. Understanding the computational capabilities of such networks, the disruptions that accompany disease and the functions of potential therapeutics would benefit greatly from network-level models that incorporate detailed mechanistic information.

Mathematical models of cell signalling exist on a spectrum in which a trade-off exists between scope and molecular detail [8-11]. The information in Bayesian nets or graphs assembled using mutual information, regression or physical association is almost entirely topological. Such models capture sets of interactions involving hundreds or thousands of biomolecules and can reveal how disease processes affect large sets of molecular [12] and cellular interactions [13]. However, such models typically include little mechanistic information and are of limited value in predicting the input–output behaviours of signalling cascades. In contrast, dynamical models, constructed using differential equations, capture detailed information on protein-protein interactions but are currently restricted to pathways involving a few dozen distinct biomolecules. We and others have described a variety of approaches to pathway modeling that attempt to combine broad scope and detailed biochemical data. They typically convert interaction networks into computable models and then train the models against experimental data [9,14-16]. Based on these models, it seems likely large-scale interaction databases represent the totality of all possible interactions that might occur between biomolecules, ignoring important cell- and context-specific differences. This arises because interaction graphs invariably contain information compiled under widely different conditions, from different cell types and even different species. When large scale, interaction-rich “prior knowledge networks” (PKNs) are converted into models and compared directly to functional data, predictive models specific to individual cell types or disease states can be constructed [9,14] in which the number of edges is significantly lower than in the starting PKN. Because missing interactions are hard to identify in this approach, it is important to assemble PKNs that adequately cover the biological process under a study; typically, this is done by hand. Manual approaches are biased and excessively restrictive in terms of the numbers of nodes and interactions however, and automated approaches to PKN assembly are clearly required.

Considerable effort has been put into collecting and collating interaction data that might be used to create PKNs for logical or kinetic modeling, but there exists no single authoritative source: information is dispersed across a multiplicity of databases that vary with respect to scope and the type of information they represent [17]. Pathguide (http://www.pathguide.org), which is intended to serve as a single point of access to interaction data involving biomolecules, links to over 300 on-line information resources [18]. High throughput experimental platforms (such as yeast-two hybrid, or tandem affinity tag-coupled mass spectrometry), result in large (and notionally “unbiased”) undirected protein-protein interaction networks (PPIN) [19] but are known to have high false positive and false negative rates, with platform-specific biases, relatively poor reproducibility, and relatively small overlap between repeats [20]. Literature-based pathway databases (Protein Signalling Networks: PSN [21]) potentially overcome this problem by capturing information recorded in thousands of papers, most of which involve mechanistic, hypothesis-driven experiments. Such data contains directional (substrate-product), and causal information (e.g. activation-inhibition relationships) and could, in principle, capture virtually the entire repository of published data on biomolecular interactions [22]; the number of interactions continues to grow as text mining algorithms get more sophisticated [23]. However, in the absence of a widely accepted semantic for describing experimental methods, automatic text mining cannot easily distinguish between co-association in text and highly specific, mechanistic information. Expert curation should, in principle, result in more reliable information, but it has recently been reported that the process is remarkably imprecise, as interactions recovered from different databases are highly discrepant [22]. Directly comparing interaction databases and combining the best features of each into a single compendium is made more difficult by the fact that existing databases were developed using different representations and formats. Standardized languages do exist (BioPAX, CellML, SBML, and PSI-MI) but none is as-yet universal [24].

In this study, we attempt to shed light on issues associated with using interaction databases as prior knowledge networks for modeling experimental data by systematically assembling and comparing pathway and protein interaction information from multiple sources. We focus on inflammation-associated signal transduction due to its ubiquity, clinical importance and extensive coverage in the literature. Inflammatory signals activate a wide range of intracellular enzymatic cascades, and many devastating diseases are directly caused by or linked epidemiologically to chronic or inappropriate inflammation; we reasoned that having accurate network resources of inflammatory pathways would be advantageous in the study of these diseases [25]. We report the compilation of interactome data involving inflammation-associated genes and interactions, and the conversion of these data into a standardized format comprising a mixed directed and undirected graph that retains resource-specific annotations, is based on Simple Interaction Format (SIF) and that can be analyzed systematically. The resulting ensemble meta-database of inflammatory networks represents a significant subset of the totality of machine- accessible human knowledge on pathways involved in inflammation. In terms of topology, the ensemble network displays the power law distribution and bow-tie architecture anticipated for signalling networks. However, we find pathway annotations to be highly inconsistent between sources, even for intensively studied pathways such as EGF signaling. It is very difficult to systematically extract focused signalling sub-networks from interaction graphs due to discrepant notation and frequent occurrence of “bypass” edges that link molecules together while skipping over essential intermediates (for example, epidermal growth factor receptor, EGFR, as a necessary component in EGF signal transduction). We therefore present a heuristic approach for utilizing interactome data that builds complexity out from linear graphs of signal transduction circuits; however, additional and more sophisticated approaches will be required if we are to effectively couple the world of large-scale interactions to functional experiments.

## Results

### Ensemble approach to network construction

We used a two-step strategy to overcome the absence of standard data formats, even among the most widely used databases in PathGuide, and thereby compile an ensemble meta-database of inflammation-associated signal transduction networks. First we compiled lists of genes involved in inflammatory signalling (nodes) from seven of the most widely used pathway databases: Cancer CellMap, GeneGo, Kyoto Encyclopedia of Genes and Genomes (KEGG), National Cancer Institute Pathway Interactome Database (NCI-PID), PANTHER, and Reactome, and a curated macrophage-specific signalling map (referred to here as the “macrophage map” [26]). In these databases, each gene is associated with one or more “pathways”. Since no unified pathway nomenclature exists, similar biological processes are associated with different pathway labels in different databases. For example "EGFR1 signalling pathway" in NetPath (Additional file 1: Table S 1, row 98) and “EGF receptor signalling pathway” in Panther (row 110) are labelled differently and therefore treated by a computer as a different pathway even though we know intuitively that they are likely to be similar.

**Figure 1:**
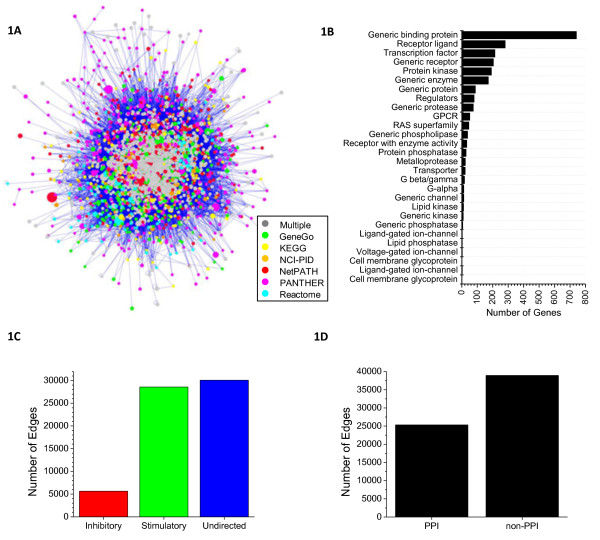
**Ensemble network of inflammatory genes and interactions.** The Ensemble network is represented as a mixed directed/undirected graph with 2,361 genes (nodes; color-coded by source, and size-coded by degree) and 63,276 non-redundant interactions (edges) **(A)**. Genes comprising the network are functionally annotated using the GeneGo ontology, and categories rank ordered by relative proportion **(B)**. Edges are functionally annotated as stimulatory, inhibitory, or undirected **(C)**, and mechanistically annotated as direct protein-protein interactions (PPI) vs. indirect (Non-PPI) **(D)**, based on a combination of annotations from the various sources.

In constructing the inflammation compendium we used broad search criteria so as to include cytokines, interleukins, chemokines, adipokines, cell adhesion molecules, extracellular matrix remodelling factors, rennin-angiotensin signalling molecules, and components of fibrogenic and angiogenic pathways. This generated a list of 2,361 genes that were components of 128 non-unique pathways (summarized in Table1 and detailed in Additional file 1: Table S 1**).** We then identified seven interactome databases, partially overlapping with the pathway databases, for which it was possible to extract machine readable interactions in Cytoscape’s SIF [27] or analogous tabular formats: a meta-database of protein-protein interactions (PPI) (Interologous Interaction Database; I2D) [28], an integrated text-mining meta-database (STRING) [29] and five of the expert-curated databases listed above (Cancer CellMap, GeneGo, NCI-PID, Reactome, and Macrophage). From these databases 63,276 non-redundant interactions were recovered (summarized in Table2). The set of 2361 genes and 63,276 interactions constituted our compendium node-edge graph (Figure1A) and is available as a gene list and an edge list in SIF format amenable to Cytoscape import (Additional file 2: Tables S 2 and S 3).

**Table 1:** Pathway databases used to extract gene lists

**Database**	**Version/date**	**Pathways**	**Inflammation**	**Genes**	**Format**
**GeneGO**	01.2010	700+	59	804	Excel Table
**PANTHER**	v6.1	165	15	1,025	SBML
**NetPATH**	01.2010	20	13	625	BioPAX/SIF
**Reactome**	v35	1081	4	173	BioPAX/SIF
**NCI-PID**	01.2010	104	28	459	BioPAX/SIF
**KEGG**	01.2010	1000+	9	564	GPML
**Macrophage**	2010	1	1	195	Excel Table
** *SUMMARY* **			** *128* **	** *2,361* **	** *Excel Table* **

**Table 2:** Pathway & interactome databases used to identify edges between genes

**Database**	**Version/date**	**Type**	**Edges**	**Graph type**
**i2D**	1.7.1	PPIN	11,327	Undirected
**STRING**	8.2.1	Text mining	35,033	Mixture
**GeneGo**	01.2010	Curated	11,994	Mixture
**Cell Map**	01.2010	Curated	12,933	Mixture
**NCI-PID**	01.2010	Curated	14,58	Mixture
**Reactome**	v35	Curated	6,930	Mixture
**Macrophage curated**	2010	Curated	504	Mixture
** *SUMMARY* **			** *64,276* **	** *Mixture* **

PPIN - protein-protein interaction network derived largely from high-throughput experiments; Mixture – both directed & signed, and undirected edges.

Genes in the ensemble graph were annotated using recognized HUGO Gene Nomenclature Committee (HGNC) IDs and colloquial names, topological properties (total and database-specific *Degree*, *Betweenness* and *Centrality*; see below), and “function” as defined by GeneGo ontology (Figure1B). Edges were labelled with the database(s) from which they were derived and all annotations derived from the source databases; edges were also classified in terms of topology as *positive*, *negative*, or *undirected* (Figure1B) and in terms of function as *direct* (protein-protein interactions including phosphorylation, binding etc.) or *indirect* (transcriptional, multi-step interactions, or undefined; Figure1C). Topological and functional features of the network can be used as filters to extract various types of relevant biological information (i.e. to model immediate early sign transduction events, one might chose to exclude all indirect interactions and transcription factors).

### Pathway mapping reveals functional topology of signalling networks

**Figure 2:**
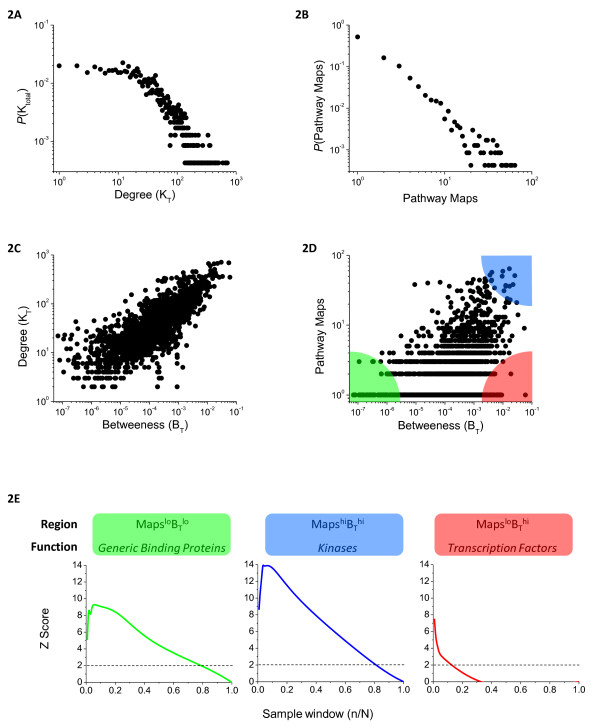
**Distribution of structural and functional annotations in the Ensemble network.** Distribution of node *Degree***(A)** and *Pathway Maps* (number of pathways a given gene is annotated as being involved in) **(B)**. These two metrics are plotted against *Betweeness* (**C** and **D** respectively) for all 2,361 genes comprising the network. The Pathway Maps vs. Betweeness distribution is separated into 3 regions; Maps^hi^B_T_^hi^, Maps^lo^B_T_^hi^, and Maps^lo^B_T_^lo^, color-coded blue, red, and green respectively **(D)**. Hypergeometric Z Scores quantify the enrichment of Kinases, Transcription factors, and Generic binding proteins across the 3 respective topological regions. Z-Scores are first computed for the top 10 genes comprising the tip of the region (n = 10), and the sample window (*n*/*N*) is then successively widened across the entire network, and scores iteratively computed to evaluate the distribution of protein function vs. topology. Z-Scores greater than 2 correspond to *P*-values less than 0.05 **(E).**

To ascertain whether the ensemble network is representative of previously analyzed interaction graphs we examined a number of information theoretic and biological properties. Complex biological and non-biological networks generally have scale-free, or power law degree distributions (where degree refers to the number of links per node). It has been proposed that this structure arises from evolutionary processes that confer robustness to random perturbations [30]. The network node degree (*K*_*T*_) for the ensemble network graph (Figure 2A) and the specific databases from which it was assembled (not shown) exhibited power law distributions although the ensemble network plateaued at the low end around degree ~10. This likely arises from our focus on highly annotated genes and multiple sources of data, resulting in a particularly dense network (average degree, *K*_*AVG*_ = 26.8) in which few nodes have few links.

The pathway annotation presented in Additional file 2: Table S 4 maps each of the 2,361 genes in the ensemble graph onto the 128 pathways from which they were derived (Tables 1 and Additional file 2: Table S 1). Many genes were included in multiple pathways and we therefore defined the metric *Pathway Maps* as the number of pathways onto which a given gene is assigned across all resources used in ensemble construction. The power law structure reappeared in the distribution of *Pathway Maps* (Figure2B), with the majority of genes (> 50%) being pathway-specific and less than 0.1% mapping onto 40+ pathways.

Signal transduction networks have been proposed to have conserved “bow-tie” structures in which a diversity of inputs converges on a limited number of central signalling nodes, which then fan out again to a diversity of downstream transcription factors and effector proteins [31]. Bow tie architectures have been identified by inspection of individual biological networks [32-37] and we wondered whether the architecture was also a present in the ensemble graph. For all nodes we therefore computed *Betweenness* (*B*_*T*_), the fraction of all shortest paths through a network that pass through a given node. Betweenness seeks to capture the importance of a node in transducing signals and is highly correlated with *Degree* (Figure2C). If a bow-tie structure holds, one would expect nodes connecting to large number of pathways to have high *Betweenness* (*B*_*T*_) and to therefore represent points of signal integration. Plotting *Pathway Maps* vs. *Betweenness* (*B*_*T*_) for all nodes, we see a positive correlation (Figure2D).

We then asked whether genes with different functions might lie in distinct regions of the Maps vs. Betweeness (B_T_) landscape. The hypergeometic Z Score (see Methods and Materials) was used to score GeneGo terms enriched in regions of the landscape corresponding to Maps^lo^B_T_^lo^, Maps^hi^B_T_^hi^, and Maps^lo^B_T_^hi^ (Figure2D). Starting at the tip of each region, we scanned across the distribution of genes and successively calculated Z-Scores for each functional protein category (Generic Binding Proteins, Protein kinases, Transcription factors; Figure2E). The graph was then divided into quadrants. We observed that “Generic Binding Proteins” were enriched in the Maps^lo^BT^lo^ region of the graph and corresponded to weakly connected genes; examples at the lowest end (*Maps* = 1, *B*_*T*_ = 0) include CCM2 (cerebral cavernous malformation 2), CD96, and CDH17 (liver-intestine cadherin). Cytosolic protein kinases were highly enriched in the Maps^hi^B_T_^hi^ region: the top ten highest scoring proteins (*Maps* ≥ 44, *B*_*T*_ ≥ 0.1%) consisted of components of the Akt and MAP kinase cascades (e.g. AKT1, MAPK1/Erk2, MAP3/Erk, SHC1, GRB2, PIK3R1/Grb1, RAF1, MAP2K1/Mek, PIK3CA, and PIK3R2) consistent with the fact that signalling kinases have many activators, many substrates, and are involved in multiple pathways. However, receptor tyrosine kinases were not enriched in the Maps^hi^B_T_^hi^ region since they correspond to inputs in the bowtie architecture. Transcription factors were highly enriched in the Maps^lo^BT^hi^ region, as they are typically pathway-specific but densely connected genes (the top 5 scoring genes in this were HNF4A (hepatocyte nuclear factor 4, alpha), POU2F1 (POU class 2 homeobox 1), TBP (TATA box binding protein), GATA1 (GATA binding protein 1), and NR3C1 (glucocorticoid receptor), all with *Maps* = 1, *B*_*T*_ > 0.47%). We conclude that the ensemble graph does exhibit topological features consistent with large-scale bow tie architecture.

### Bow tie architecture results in functional clustering of pathways

**Figure 3:**
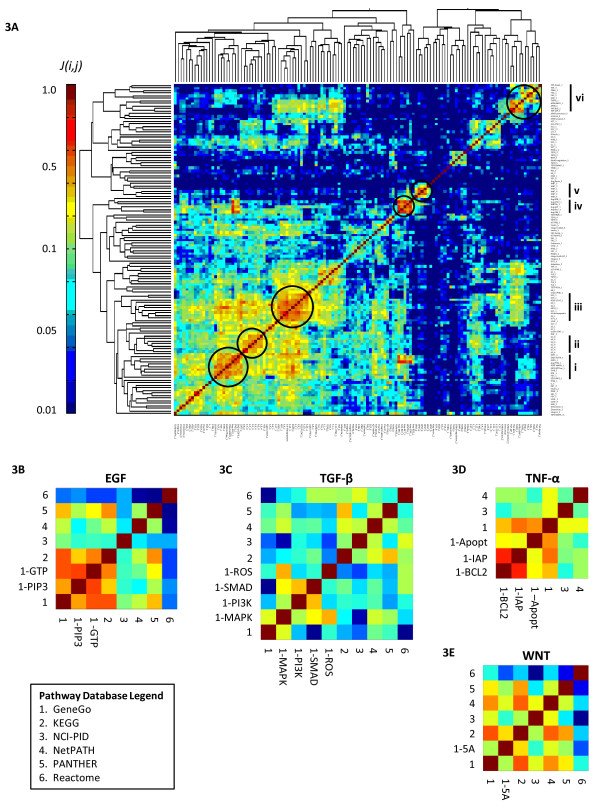
**Pair-wise similarity matrix of literature-defined pathways.** The *Jaccard index* (a similarity metric) between all pairs of 128 pathways represented as a hierarchically clustered heatmap **(A)**. For brevity, source databases are indicated by number: GeneGo [1] KEGG [2], NCI-PID [3], NetPATH [4], PANTHER [5], and Reactome [6]. The GeneGo database [1] sub-categorizes pathways based on the use of alternate downstream effectors, as indicated in the labels. 7 dense pathway clusters are highlighted via black circles and labelled I through VII. Focusing on 4 extensively studied pathways (EGF, TGF-β, TNF-α, and Wnt; **B** through **E** respectively), Jaccard index matrices for the same pathway as defined in different database sources. Note the high level of discordance between alternate sources.

Many pathways share common signal processing elements and we wondered whether this property was captured in the ensemble network. As a corollary to the gene-centric *Pathway Map* metric, we use the *Jaccard Index* (*J(i,j)*), a metric of set similarity, to quantify the fraction of genes common to two pathways *i* and *j* (see Methods and Materials). Computing this pair-wise metric for all 128 pathways produced a square symmetric matrix, represented as a heatmap in Figure3A, with rows and columns organized via unsupervised hierarchical clustering. One would intuitively expect pathways with many common elements to have functionally similar phenotypic annotation, a phenomenon that has been demonstrated previously in a limited and focused manner [38]. We confirmed that this was true in a larger sense: functionally related pathways clustered together with respect to the components they contained, as revealed by dense orange/red regions along the diagonal. Six distinct clusters are highlighted by way of illustration and labelled as I through VI. Cluster I represent a set of growth factor Receptor Tyrosine Kinases (RTKs for EGF, FGF, HGF, PDGF, and Endothelins) that share many components, particularly the PI3K/Akt and MAP kinase cascades (e.g. PI3KCA, AKT1, MAKP1, MAP2K1, HRAS, GRB2); many of these pathways converge on the oncogenic transcription factor ELK1. Cluster II represents Interleukins IL2-9 that co-activate the PI3K/Akt, MAPK Kinase and JAK/STAT cascades (e.g. MAPK1, AKT1, JAK1, STAT3). Cluster III is an intriguing mix of interleukins and RTK ligands (IGF, VEGF, PDGF, LEP, IL4, IL2, IL9, IL10, IL17, IL23) that fall together because they activate both PI3K/Akt and NFκB pathways (e.g. PI3KCA, AKT1, NFKB1, RELA). Cluster IV consists of renin-angiotensis signalling events (ERK, STAT, AKT, ROS-dependent) and WNT5A; these pathways have in common an ability to activate phospholipase Cβ (PLCB1-4). Cluster V consists of the WNT pathway which is annotated differently in various databases but always includes wingless ligands (WNT1-3, 5A, 7A) frizzled receptors (FZD1-9), low density lipoprotein receptor-related protein 5 (LRP5), and intracellular signalling molecules such as glycogen synthase kinase 3 beta (GSK3B) and β-catenin (CTNNB1). Cluster VI contains pro-apoptotic death ligands (TNF, TRAIL, FASL, and APRIL/TNFSF13) that share an interaction with TNF receptor associated factors (TRAF2,3), Fas -associated death domain (FADD), Bcl-2 (BCL2), and caspase 8 (CASP8). Despite problems with inconsistent nomenclature, we conclude that clustering pathways by the *Jaccard Index* uncovers core signal transduction cascades shared between differing pathways. The analysis provides further evidence of a bow tie structure, in that diverse extracellular ligands activate combinations of a few highly conserved cascades (PI3K/AKT, MAPK/ERK, JAK/STAT, NFKB, and apoptotic cascade). Finally, the data suggest interesting differences and similarities among receptors: clustering of growth factors (EGF, FGF, HGF, PDGF) with each other is expected, but inclusion of Endothelins, which function via G protein-coupled receptors, is less obvious as is co-clustering of six interleukins with growth factors such as IGF, PDGF etc. Further analysis of similarities in network properties involving these proteins may uncover whether co-clustering arises from physiologically meaningful cross-talk.

While many databases contain representations of “canonical” signalling pathways, it is not clear how consistent the definition of pathways is. To examine this we focused on 4 extensively studied, and presumably well-defined signalling pathways lying downstream of Epidermal Growth Factor (EGF), Transforming Growth Factor-β (TGF-β), Tumour Necrosis Factor-α (TNF-α), and Wingless (Wnt; Figure3B-E). We observed remarkably poor agreement (consistently less than 10%) among different databases regarding pathway components. Note that the GeneGo database sub-categorizes some pathways based on the use of alternate downstream effectors (e.g. TGF-β signalling into ROS, SMAD, PI3K, and MAPK-dependent branches), and these sub-categorizations are in closer agreement than the pathways as defined in different sources. Thus, what constitutes a “canonical” pathway is database specific. This inconsistency in annotation may reflect underlying biology, in that signal transduction events are often context-dependent, or it may reflect the absence of a controlled vocabulary (as noted above). Regardless, such complexities are rarely accounted for in databases (except perhaps the macrophage PSN) or in large-scale analysis of protein networks. This raises a significant problem for mechanistic modeling, since in the absence of objective measures of database bias or reliability it is not clear which genes/proteins to include for modeling or experimental measurement.

### Variable pathway annotation is a significant contributor to inconsistencies between interactome databases

**Figure 4:**
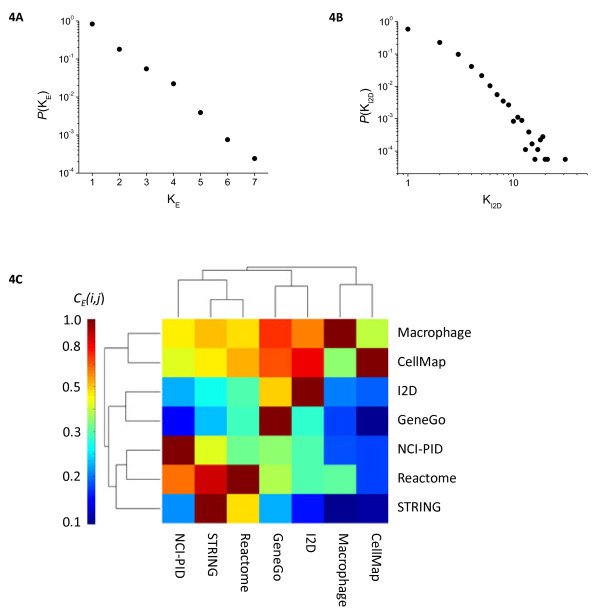
**Edge consistency between interaction databases.** Distribution of *Edge Weights* (the number of databases an interaction is found in) for all 63,276 interactions **(A)**, and I2D-specific edge weights (the number of PPI datasets a PPI is found in) for 11,327 interactions form the I2D meta-database **(B)**. *Fractional Edge Overlap* scores (proportion of interactions consistent beteen 2 databases) are represented as a hierarchically clustered heatmap **(C)**. Note the heterogeneity, and overall poor concordance between different sources.

Inconsistencies across databases with respect to which genes lie in which pathways led us to examine the consistency with which molecular interactions (edges) were present among 7 different interaction databases. We defined the *Edge Weight* (*K*_*E*_) as the number of databases in which a specific interaction from the ensemble graph was present in each of the databases from which it was assembled. We observed that *K*_*E*_ followed a power-law relationship across 63,276 interactions with the majority of interactions (> 80%) specific to one database and fewer than 0.1% appearing in ≥ 6 databases (Figure4A). Performing a similar analysis on a compilation of protein-protein interactions derived from multiple high-throughput sources (the Interologous Interaction Database - I2D) in with the Edge Weight (*K*_*I2D*_) is defined as the number of protein-protein interaction databases containing a given revealed a similar distribution (Figure4B). Inconsistency with respect to the inclusion of edges is therefore a conserved feature of both pathway and interaction databases [20,39].

To analyze this issue further we focused on the degree of agreement in edges among the 7 interactome databases. The *Edge Consistency* (*C*_*E*_) was defined as the fraction of edges in database *i* also found in database *j* and involving genes shared between the two databases (see Methods and Materials). Performing pair-wise analysis produced a square, non-symmetrical matrix represented as a hierarchical clustered heatmap in Figure4C (x- and y- axes corresponding to databases *i* and *j*). We observed a wide range of consistencies among pairs with values ranging from 16% to 82%. Edges from the curated Macrophage and CellMap databases were the most consistent (*C*_*E*_ >50%) with the other five databases in the analysis. Paradoxically, Macrophage and CellMap were not very consistent with each other (*C*_*E*_ = 6.6 and 15%). This may reflect differences in the cell types under consideration: macrophages in the Macrophage PSN and tumor cells in CellMap. Edges from the NCI-PID and Reactome databases were also fairly consistent across sources (30% and 43% respectively) but the most comprehensive interactome databases (GeneGo, I2D, and STRING) were significantly less so (21- 31%). By comparing *Edge Weight* and *Edge Consistency* we conclude that the most significant source of inconsistency among databases involves the ways in which pathways are annotated and gene sets assembled; this is primarily a failure of biological understanding rather than computational procedures and emphasizes the importance of bringing more data to bear on network maps.

### Canonical pathway annotations represent “fuzzy” modules

**Figure 5:**
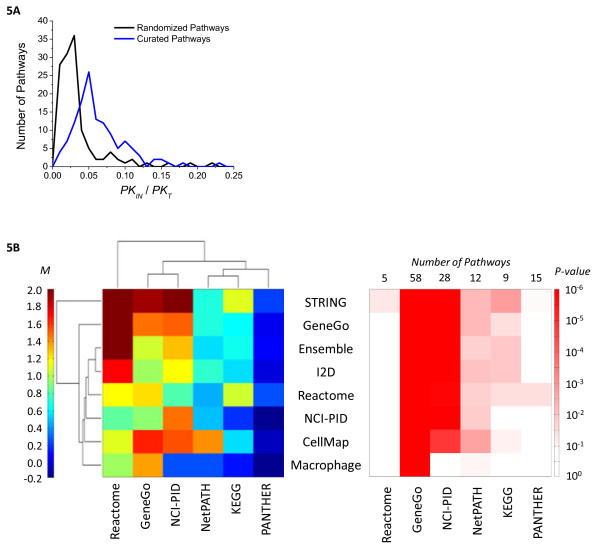
**Pathway modularity.** Fraction of internal edges (within pathway; *PK*_*IN*_/*PK*_*T*_) for 128 curated pathways (blue) vs. the distribution as expected by chance for randomized pathways (black) **(A)**. The shift is statistically significant (*P* < 10^-18^) indicating a high level of modularity on average across pathways. **(B)** Average modularity (*M*; see Methods for mathematical definition) of pathways derived from the 6 pathway databases (columns) using edges derived from the 7 interaction databases plus the Ensemble network (rows), represented as a hierarchically clustered heatmap. **(Bii)** Corresponding P-values based on the Mann–Whitney *U*-Test. Note the heterogeneity; some pathway databases are highly modular regardless of interaction source, while others are less so.

It is widely claimed that biological networks exhibit modularity [40] but it is not clear whether modularity can be discerned in large network graphs. To address this question, we defined the *Total Pathway Connectivity* (*P*_*T*_) for each of the 128 annotated pathways in our ensemble graph as the sum of all degrees (*K*) for genes included in a pathway. This can be divided into the total degree of *internal edges* (*P*_*IN*_), which connect 2 genes within a particular pathway and the total degree of *external edges* (*P*_*EX*_) that connect genes inside and outside a pathway. *P*_*IN*_ and *P*_*EX*_ correspond roughly to “canonical” vs. “cross-talk” interactions. The simplest definition of a network module is a group of nodes in which the number of internal interactions is greater than the number of external interactions (*P*_*IN*_/*P*_*T*_ ≥ 0.5) [41,42]. More mathematically complex definitions have been proposed but they are limited in that they assume a given node is assigned exclusively to one module [43,44] a property that is inconsistent with inclusion of many genes in in multiple pathways (*Pathway Maps* > 1; e.g. AKT1, MAP2K1, STAT3, NFKB1). We therefore computed the fraction of internal edges for each pathway (*P*_*IN*_/*P*_*T*_) and compared it to the value expected by chance (*E*_*IN*_/*E*_*T*_) given a random assignment of genes to pathways (Figure5A). The distribution of *P*_*IN*_/*P*_*T*_ across the 128 pathways was significantly right-shifted compared to the randomized control (*P* < 10^-18^) implying that literature-defined pathways in the ensemble graph display a higher degree of modularity than would be expected by chance alone. However, *P*_*IN*_/*P*_*T*_ peaked at ~5% and had a maximum value of ~25%, implying that none of the literature defined pathways met the simplest definition of modularity (*P*_*IN*_/*P*_*T*_ < 0.5). Thus, the vast majority of interactions in the ensemble network constitute “cross-talk”.

It seemed possible than the process of creating an ensemble network might obscure modularity found in individual databases. We therefore asked whether the degree of modularity differs between databases. We defined *Pathway Modularity* (*M*) as a metric to compare observed modularity (*P*_*IN*_/*P*_*T*_) to what would be *expected* by chance (*E*_*IN*_/*E*_*T*_) (see Methods and Materials); *M* = 0 corresponds to a random distribution of internal and external edges, while *M* = 1 corresponds to two-fold more (%100 increase) internal edges than would be expected by chance. The modularity *M* was calculated for pathways as defined in each of the 6 pathway databases based on interactions drawn from the ensemble network or from 7 constituent interaction databases. The resulting 6x8 matrix is represented as a hierarchically clustered heatmap in Figure5Bi, and the statistical significance of the metric is represented as corresponding matrix of *P*-values in Figure5Bii.

We observed that pathways and interactions derived from the same database consistently showed high *M* scores (1.15 to 1.48) but the highest values for *M* were observed when pathways and interactions were drawn from different databases. For example GeneGo pathways display a higher value for *M* on interactions drawn from CellMap and STRING (*M* = 1.65, 1.91) than on GeneGo interactions (*M* = 1.47); Reactome pathways had higher Modularity on STRING, GeneGo, and I2D interactions (*M* = 1.71 to 4.85), than on Reactome (*M* = 1.15). Moreover, the pattern of clustering clearly reveals large differences in Modularity between databases. Modularity (*M*) of GeneGo and NCI-PID pathways are particularly significant (*P* < 10^-14^ and 10^-5^ respectively, corresponding to *P*_*IN*_*/P*_*T*_ values of 1.7 and 1.6), while PANTHER pathways display essentially no significant degree of modularity (*P* = 0.04 to 0.97, corresponding to *P*_*IN*_*/P*_*T*_ values of −0.14 to 0.26). Compared to the ensemble network (*P* = 10^-18^ at *P*_*IN*_*/P*_*T*_ = 0.05) these are very broad range of values. Differences in *Modularity* score between databases may reflect different areas of emphasis and different types of curation. For example, the NCI-PID database (*M* = 1.48) was developed specifically around protein signal transduction, for which we might expect significant modularity, while NetPATH and PANTHER include downstream transcriptional circuits which are probably more interwoven. We conclude that individual pathway databases exhibit “fuzzy” modularity [45] that is statistically significant but still involves many “cross-talk” interactions.

### Representation of mechanism in the ensemble network

**Figure 6:**
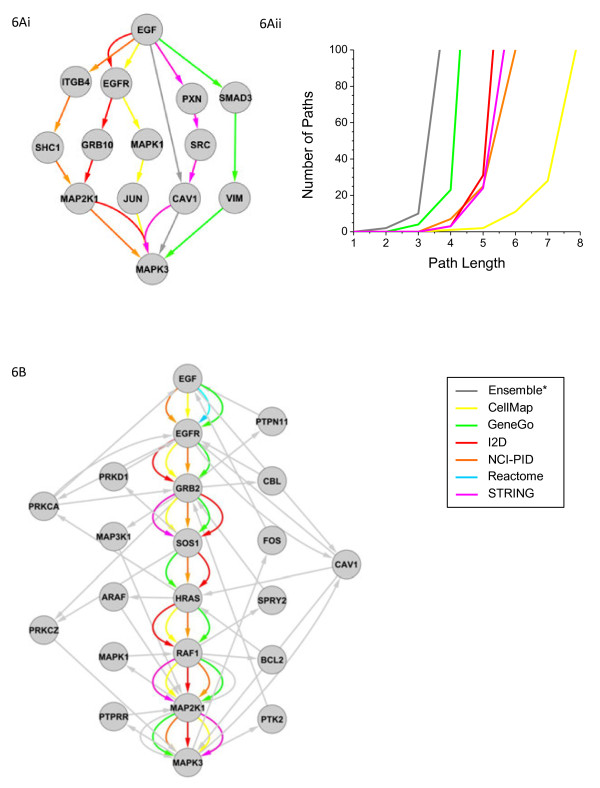
**Ensemble representation of EGF signalling network.** Alternate signal transmission routes connecting the extracellular ligand EGF to the sentinel marker of pathway activity MAPK3 (ERK). Shortest paths **(Ai),** and total number of paths as a function of path length **(Aii)**, color-coded by database source. **(B)** The “consensus” linear EGF pathway, with edges color-coded by source, plus additional connections through the ensemble network color-coded grey. Note the Ensemble network edges are derived from multiple sources, with all connections in the consensus pathway represented in at least 3 different databases.

A key goal of this paper was to create a prior knowledge network that would represent a relatively unbiased starting point for kinetic or logic-based modeling of inflammatory and receptor-mediated signal transduction. To see whether this might be possible starting with the ensemble graph, we focused our attention on EGF, EGF receptor, and downstream cytosolic signalling proteins; the EGF pathway is clinically important and has been subjected to extensive experimental and computational analysis [46-51]. Gene sets comprising EGF signalling from 6 of the pathway databases were compiled, and directed protein-protein interactions (protein binding, phosphorylation, etc.…) were mapped from the 7 interactome databases. We limited the analysis to directed edges, as these are the most helpful in building mechanistic models involving enzyme-substrate relationships. To identify sets of interactions linking the extracellular ligand EGF to the intracellular kinase MAPK3 (ERK), a key step in immediate-early signal transduction, we searched for shortest paths connecting EGF to MAPK3 in each of the 6 interactome databases and the Ensemble network (Figure6Ai). Ensemble paths comprise edges that can derive from multiple databases; EGF → CAV1 from NCI-PID, and CAV1 → MAPK3 from GeneGo, for example. Edges contained in the shortest paths for specific databases were in many cases also found in other databases. Focusing on GeneGo for example, the EGF → SMAD3 edge is also present in the NCI-PID database, and the SMAD3 → VIM and VIM → MAPK3 edges are both present in the i2D database. However, these details have been omitted for clarity.

A remarkably large number of paths are present in the ensemble database linking EGF and MAP3K: the precise number depends on how long a path is considered (see below). Both the CellMap and NCI-PID databases correctly show EGF binding to its canonical receptor EGFR in the shortest paths, but they also suggest that the ligand can interact directly with ITGB4 (integrin-β4), PXN (Paxillin), SMAD3, or CAV1 (caveolin-1). While there are indeed likely to be multiple routes by which signals from EGF are propagated to downstream regulators, is not plausible that every route represented in the ensemble database actually exists. Moreover, since all of known biological activities of EGF require binding to transmembrane ErbB receptors (EGFR is identical to ErbB1), the existence of direct interactions between EGF and ITGB4 and other intracellular proteins is highly improbable. Instead, these molecules are known to be phosphorylated/dephosphorylated via kinases and phosphatases activated by EGF, including MAPK [52], SHP2 [53], ERK [54], and EGFR [55] respectively. Short pathways (ranging from 2 to 4 steps) that omit the requirement for EGFR in EGF activity or RAS in MAP3K activation are examples of “bypass interactions,” interactions that are present in network databases but that are biochemically highly implausible.

To determine the frequency of such potential “bypass interactions” we asked how many different routes link EGF to a downstream node. In general this is an NP-hard problem, but, by setting an upper limit on path length the problem is computationally tractable. We calculated the total number of alternate paths connecting EGF to MAPK3 for the ensemble and each constituent database for path lengths of 1 through 8 (Figure6Aii). The number of alternate routes was observed to increase exponentially with path length. At a path length of 4, there existed 146 alternate routes connecting EGF to MAPK3 via the Ensemble network, and within 8 steps, > 100 alternate paths are found in all databases. Mechanistic considerations suggest the actual path length to be ~8 steps corresponding to ligand-receptor binding, assembly of intracellular signaling complexes, activation of Ras, followed by phosphorylation of Raf, MEK and then Erk (MAPK3; http://en.wikipedia.org/wiki/MAPK/ERK_pathway; Figure6B). The fraction of paths that pass through EGFR (and are therefore biochemically plausible) varies by path length, but for the ensemble this stabilizes at approximately 20%. We can thus definitively classify at least 80% of the alternative pathways as being bypass edges, and thus of little utility in a mechanistic model. To ensure these results were not unique to the EGF signalling network, we performed the same analysis on the 3 pathways analyzed in Figure3C-E; TGFB, TNF, and WNT, using SMAD4, NFKB1, and GSK3B as downstream sentinel nodes (Additional file 1: Figure S 1). The results were similar in all cases; while canonical receptors and pathway components are recurrently identified, many other pathways linking ligands to downstream transducers were found in all databases and the total number of paths increased exponentially with path length.

It is not obvious how we should discriminate computationally among different representations of EGFR signalling without imposing prior knowledge. If we consider the probable origins of many of the bypass interactions in the EGF sub-network we can appreciate why they are difficult to eliminate: a paper claiming that EGF activates SMAD3 does not necessarily mention the involvement of EGFR since this information is implicit. Indeed, it is probable that the more nearly canonical a biochemical step (EGF acts via EGFR), the less likely it is to be mentioned in the contemporary literature. Thus, some of the highest likelihood interactions are underweighted in databases even though the statement “EGFR activates SMAD3” in no way contradicts the claim that EGFR is a required intermediary. Because of the large number of alternative routes, we conclude the signalling pathways as they are commonly understood in the literature are topologically non-identifiable in interaction databases given current experimental technologies and practices. While computational methods are available for reducing network complexity based solely on topology [56], we do not yet have the large-scale experimental data sets needed to weed out the direct interactions from the indirect ones in graphs such Figure6Ai; indeed, even when the methods are available [9,57] it is not obvious whether it is worth spending the effort simply to rediscover highly studied mechanisms.

In an attempt to build a network for EGF signalling rich in relevant interactions, we sought to merge network information with a more conventional linear map of signal transduction. We first defined an EGF minimal scaffold as the generally accepted reaction sequence EGF → EGFR → GRB2 → SOS1 → HRAS → RAF1 → MAP2K1 (MEK) → MAP3K (ERK), while recognizing that there is demonstrably more complexity to this pathway, particularly at the level of receptor and MAPK signalling. All scaffold interactions were found in > 3 databases, significantly enriched for high weight edges (*P* = 2.7 × 10^-8^, hypergeometric test). We then used the scaffold to identify interactions between successive nodes (Figure6b). We sought a minimal expansion in the map by searching for links between these components separated by one intermediate node. 14 additional molecules were identified, mediating multiple feed-back and feed-forward loops, as well as potential bypass interactions. These also serve as points of “cross-talk” to other pathways, including growth factors (i.e. VEGF, PDGF, FGF, HGF, WNT, TGFB), chemokines, interleukins, interferons, death receptors (FAS, APRIL), cell adhesion, insulin, and toll-like receptors (TLR) (see Additional file 2: Table S 4 for complete pathway maps). In many cases, these new interactions make sense: Fos is activated by EGF and is plausibly represented as being part of a feedback pathway, Protein kinase C is also activated by EGF, and multiple MAPK family members are involved in regulation of MAPK3. This approach, in which a seed scaffold is first defined and then a series of interactions 2 or more links out added to build out a more complete network may serve as a heuristic approach to adding complexity in a step-wise manner to linear pathway models. An automated approach to performing such an expansion starting with a seed and adding interaction data has recently been described [58] and could presumably be extended to include a filter for particularly problematic bypass edges (ligands acting without receptors for example).

## Discussion

The goal of this work was to create and analyze an ensemble database that represents the superposition of machine-readable knowledge on the topologies of inflammatory networks in humans as a prelude to more detailed network analysis and mathematical modeling. Different subsets of on-line resources can be queried for gene names and interactions and we show that it is possible to combine this data into a single SIF-compliant protein interaction network rich in information and amenable to Cytoscape import. Interactions vary in type with some directed and signed and others undirected and unsigned, depending on the source of data. The average number of interactions per node in the ensemble network is high (degree ~27) and it displays a power law degree relationship. The ensemble network also exhibits evidence of a bow-tie structure in which a multiplicity of pathway-specific receptors feed into a smaller set of highly interconnected intracellular kinases and signalling molecules which then output onto a larger collection of pathway-specific transcription factors and effectors. Overall, genes from 128 pathways are present in the final network but the majority of genes (> 50%) are pathway-specific with fewer than 0.1% mapped onto 40+ pathways. The set of highly represented genes includes many of the cytosolic kinases lying in the middle of the bow-tie structure (PI3K/AKT, MAPK/ERK, JAK/STAT, NFκB).

A striking feature of the databases from which the ensemble was assembled is that they are highly inconsistent with respect to the number of nodes and the number and identities of the interactions for a given node. For example, more than 80% of the interactions in the ensemble were specific to one database and fewer than 0.1% appeared in six or more databases (the remaining exhibited a power-law relationship to frequency). We find the root of this inconsistency to lie in the wide discrepancy in pathway annotations between databases. Even when we focused on highly studied pathways activated by EGF, TGF-β, TNF-α, and WNT ligands, we observed remarkably poor agreement (consistently less than 10%) regarding the constituents. What constitutes a “canonical” pathway therefore appears to be database (or even expert) specific. Both at the biochemical and phenotypic level, exogenous stimuli are known to exhibit profound cell- and context-specific effects [59]. Discrepancies in pathway annotations between databases may be reflective of this [22], but it is currently impossible to determine whether the primary problem is real biological variation, the absence of suitable controlled vocabularies or another technical problem.

Given extensive discussion about the “modularity” of biological networks [60] we asked whether the ensemble graph or the databases from which it was assembled show evidence of modularity. The simplest way to define a module is as a set of genes for which interactions among genes within the set is more frequent than interactions with genes outside of the set. Under these circumstances we observed that only 5% of edges in the ensemble network constituted intra-pathway interactions and the vast majority of interactions therefore crossed pathways (potentially representing sources of “cross-talk” and consistent with data arising from high-throughput interaction screens for components of MAPK, TGF-β, and TNF-α pathways [4-7]. Four obvious and non-exclusive explanations for this data suggest themselves: (i) biological pathways represented in the ensemble database are not modular in any meaningful sense and instead comprise closely connected networks (ii) we cannot easily identify modularity in large networks through pathway annotation because the definition of these pathways is highly subjective and variable from one database to the next (iii) the ensemble network contains many interactions that do not exist in reality (iv) modularity can only be understood with respect to specific temporally-restricted biological functions. The later possibility is the most interesting: while it is true that the MAP kinase cascade can be considered to be a component of a relatively well-defined enzymatic pathway that transduces signals from growth factor receptors to the cell nucleus, the organization of this cascade changes over time as receptors adapt and negative regulatory pathways are activated. Moreover, in cells exposed to a different growth factor, activation of the MAP kinase cascade can have very different biological consequences.

While the degree of Modularity among the 128 pathways annotated in the ensemble network may be low, it is statistically significant compared to what is expected by chance. This may constitute a form of “fuzzy” modularity, wherein diffuse and overlapping modules are integrated with one another and the broader cellular network, perhaps conferring flexibility in adapting to complex environmental perturbations [45]. Moreover, we observe a wide range in our statistical metric of modularity between pathway databases (ranging from *P* < 10^-14^ for GeneGo, to an average of *P* = 0.5 for PANTHER) reflecting the widely different curation standards. Results emerging from cancer genome sequencing projects validate the concept of pathways as functional modules. For tissue-specific and even clinically homogeneous cancer subtypes, thousands of diverse mutations have been catalogued. However, the majority of mutations can be mapped onto a limited number of canonical signal transduction pathways (TP53/RB1, PI3K/AKT, Wnt, Hedgehog, and TGF-β). Moreover, mutations within the same pathway are often functionally equivalent (exclusive), and specific combinatorial patterns of pathway activation/deactivation are required to induce transformation [61-63]. Viewing pathways as functional modules is thus a useful concept for integrating diverse molecular data and reducing biological complexity to simpler principles. The trick will be to learn to identify these modules in interaction graphs, perhaps by implementing automated network module detection algorithms [42], and comparing how such *a priori* defined modules overlap with annotated pathways.

Can pathway and interactome databases be used as tools for modeling functional experiments in specific cell types? Currently pathway databases are employed largely to generate static network maps for topological analysis and, with high-throughput genomic, data to assist in the identification of meaningful co-variation [64]. Increasingly, however, it is becoming recognized that computable models are crucial for the quantitative analyses of biological systems. The utility of computable models arises from their ability to making predictions that can be tested experimentally. A reasonable approach to building computable input–output models would involve assembling a comprehensive scaffold of molecular interactions, converting the scaffold into one or more models and then comparing the models to various types of experimental data [10]. Qualitative formalisms such as Boolean logic appear to be effective in this role [9]. Moreover, by focusing on relatively restricted portions of interactions networks, it should also be possible to inform kinetic models of mass-action biochemistry [50,65]. In both cases, it is necessary to start with complete topologies [66] and both errors and omissions have profound implications for experimental design, data analysis, and model development.

Using four exemplary signalling systems (EGF, TGFB, TNF, and WNT), we show that downstream signalling kinases are connected to extracellular ligands via hundreds of alternative topologies, many of which are biochemically implausible in that they do not involve transmembrane receptors or the known topology of MAP kinase cascades. We refer to these as “bypass” edges. A number of algorithms are available for reducing such network redundancies and idiosyncrasies using topology alone [56] or using experimental data [67]. These may represent a tractable way to initiate model topologies in the absence of expert prior knowledge. We illustrate an alternate heuristic approach for utilizing interactome information to building out network complexity from simple linear scaffold. Nonetheless, it is clear that additional research is required in this area.

## Conclusions

In summary, we have identified wide-ranging discrepancies in how signalling pathways are defined between different databases both with respect to molecular components (nodes) and interactions (edges). Such discrepancies are likely to arise both from biological factors, such as the context-specific nature of cellular signal transduction, and also technical problems such as the absence of controlled vocabularies for defining what constitutes a particular pathway. In addition, because of the way they are constructed, interaction databases contain large numbers of bypass links that omit essential molecular dependencies, such as a requirement for receptors in transducing the activities of extracellular ligands. As a result, it is difficult to identify well-known signal transduction pathways from interaction databases. However by starting from small “textbook” scaffolds, interaction databases can be used to successively build out network complexity in a step-wise manner. This generates the prior knowledge networks from which cell and disease-specific models can then be deduced using experimental data.

## Methods

### Ensemble network construction

The 6 Pathway databases listed in Table1 [GeneGo (http://www.genego.com/), PANTHER (http://www.pantherdb.org), NetPATH (http://www.netpath.org), Reactome (http://www.reactome.org), the National Cancer Institute Pathway Interaction Database (NCI-PID; http://pid.nci.nih.gov), and the Kyoto Encyclopaedia of Genes and Genomes (KEGG; http://www.genome.jp/kegg)] were searched for inflammation-associated pathways, with inclusion criteria based on extensive literature curation of ligands and processes involved in chronic inflammatory diseases (Additional file 2: Table S 1). Gene lists (Entrez IDs) for pathways meeting our criteria were downloaded and compiled into a master list, while maintaining their sources (Additional file 2: Table S 4). Gene Symbols and official Identifiers were derived from the HUGO Gene Nomenclature Committee (HGNC) database (http://www.genenames.org/aboutHGNC.html), and the GeneGo ontology was used to annotate protein functions (Additional file 2: Table S 2). Human-specific Interactions between genes in the master list were identified by searching the GeneGo, I2D v1.7.1 (http://ophid.utoronto.ca/ophidv2.201), and STRING v8.2 (http://string-db.org/) databases. The Cancer Cell Map (CellMap: http://cancer.cellmap.org/cellmap), NCI-PID, and Reactome pathways were downloaded as SIF files from Pathway Commons (http://www.pathwaycommons.org/pc/). While many additional similar databases exist, these arguably represent the most widely used, and by extension presumed highest quality resources available. All of the databases considered represent long-term collaborative efforts, maintained by full-time staff, regularly updated, and supported by large academic institutes as well as a commercial organization. GeneGo (Thompson Reuters) is the sole commercial, pay-for-access database. This was included so as to cover both publicly available and commercial resources. In addition, A manually-curated network of molecular interactions involved in macrophage activation (Macrophage) as defined in [26] was downloaded from the Supplementary Information of Raza et al. Molecules in these networks were converted to their respective Entrez Gene IDs and filtered for genes in the master list. Interactions involving molecular complexes were combinatorially expanded to pair-wise gene interactions, and only direct gene/protein interactions were considered. The 7 interaction sources were integrated as the Ensemble network (Additional file 2: Table S 3). The network was visualized in Cytoscape v2.8 (http://www.cytoscape.org).

### Structural analyses

The *Jaccard Index* measures similarity between sample sets, defined as the size of the intersection divided by the size of the union of the sample sets. The *Jaccard Index* between pathways *i* and *j* (*J*(*i,j*)) is thus defined as:

Where *n(i,j)* is the number of common genes, and *N(i)* and *N(j)* are the total number of genes in pathway *i* and *j* respectively.

Similar to the Jaccard index, but accounting for vast differences in database coverage, we define the *Edge Consistency* (*C*_*E*_*(i,j)*) between databases *i* and *j* as :

Where *e(i,j)* is the number of common edges, and *E(i)*is total number of Edges in database *i* between genes also present in database *j*.

Betweeness centrality of a node is defined as the fraction of all shortest paths in a network that pass through it. The Betweeness centrality for all genes in the Ensemble network was computed using the *NetworkAnalyzer* Cytoscape plugin (http://med.bioinf.mpi-inf.mpg.de/netanalyzer/).

### Functional enrichment analysis

To examine the enrichment of protein functions within topological regions of the ensemble network, we used the hypergeometric Z-score, an easily computable metric for assessing gene set enrichment [68]. The hypergeometric Z-score is defined as:

Where *N* = total number of elements (genes), *R* = total number of positive elements (protein functional categories), *n* = sample size, and *r* = number of positive elements in the sample.

The metric is derived from the standard, or Z-score:

Substituting the average (*μ*) and standard deviation (*σ*) for the Hypergeometric distribution:

Values greater or less than ±2 thus approximately correspond to p-values ≤ 0.05.

Genes comprising the network are first rank ordered, in this case based on *Pathway Maps* &*Betweeness* scores. Starting at one end of the ranked gene list (either Maps^lo^B_T_^lo^, Maps^hi^B_T_^hi^, or Maps^lo^B_T_^hi^) a sample (size *n*) is assessed for enrichment of a categorical classification (in this case, protein function designations Generic Binding Proteins, Kinases, and Transcription Factors). The *sample window* (*n*/*N*) is then successively widened across the gene set, and the score iteratively computed, eventually covering the entire set of genes. The Z-score can was plotted as a function of the *sample window* size (*n*/*N*), to examine the distribution of functional categories within the network.

### Pathway modularity

The Total Pathway Connectivity (*P*_*T*_) is defined as the sum of all pathway gene degrees (*K*);

For *n* total genes associated with pathway *i*. Edges comprising the Total Pathway Connectivity (*P*_*T*_) can be divided into Internal (*P*_*IN*_) vs. External (*P*_*EX*_), defined as edges which connect 2 genes within a pathway vs. edges which connect a gene within the pathway to a gene outside of the pathway.

Pathway Modularity (*M*) is defined based on the ratio of internal to total pathway edges (P_IN_/*P*_*T*_) compared to what would be expected by chance, given a completely random association of genes to pathways. The expected ratio of internal to total pathway edges (*E*_*IN*_/*E*_*T*_) for each of the 128 pathways was determined by randomizing the gene lists while maintaining the Pathway Maps for each gene. The number of internal and total edges were then counted for 100 randomizations, and the average values taken as the expected ratio (*E*_*IN*_/*E*_*T*_). These calculations were performed using the Ensemble network, as well as the 7 interaction database-specific networks. Pathway Modularity is then given by:

Statistical significance of the Pathway Modularity scores for each of the 6 pathway databases were assessed by comparing the (*P*_*IN*_/*P*_*T*_) and (*E*_*IN*_/*E*_*T*_) distributions using the non-parametric Mann–Whitney *U* test.

### EGF pathway identification

The shortest paths connecting EGF to downstream constituents were identified using Dijkstra’s algorithm, and total number of paths computed using an iterative depth-first search.

All analyses were performed using MATLAB R009b software (The Mathworks, Natick, MA).

## Competing interests

The authors declare that they have no competing interests.

## Authors’ contributions

DCK conceived of the study and conducted all analyses. DCK and PKS wrote the manuscript. JSR, JS, JMB, and DAL contributed key ideas and critically revised the manuscript. All authors read and approved the final manuscript.

## Supplementary Material

**Additional file 1: Figure S1** Ensemble representation of TGFB, TNF, and WNT signalling networks. Alternate signal transmission routes connecting the extracellular ligands TGFB1 **(A)**, TNF **(B)**, and WNT **(C)** to their respective sentinel markers of pathway activity, SMAD4, NFKB1, and GSK3B. Shortest paths **(i),** and total number of paths as a function of path length **(ii)**, color-coded by database source. Click here for file

**Additional file 2: Table S1** List of Pathways. **S2** Gene Annotations. **S3** Edge Annotations. **S4** Pathway Gene sets. Click here for file
