# Supplementary figures and images for: Creating and analyzing pathway and protein interaction compendia for modelling signal transduction networks
Source: BMC Syst Biol. 2012 May 1;6:29. doi: 10.1186/1752-0509-6-29 (PMC3436686; doi:10.1186/1752-0509-6-29)

S1Ai

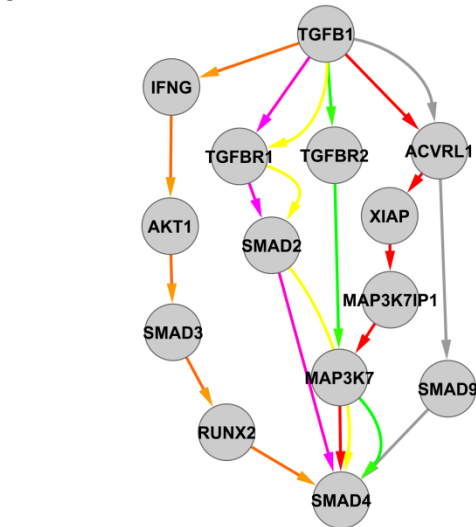

S1Aii

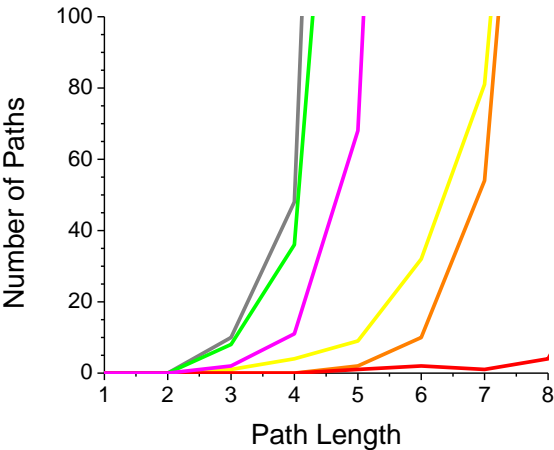

S1Bi

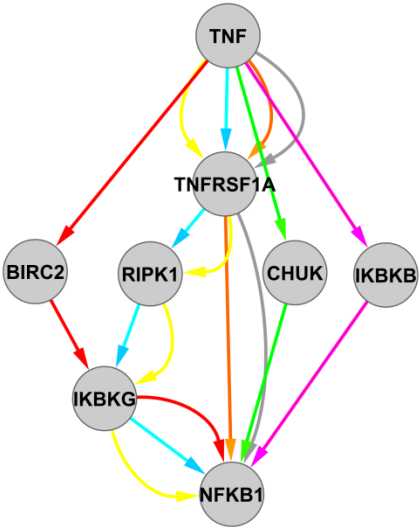

S1Bii

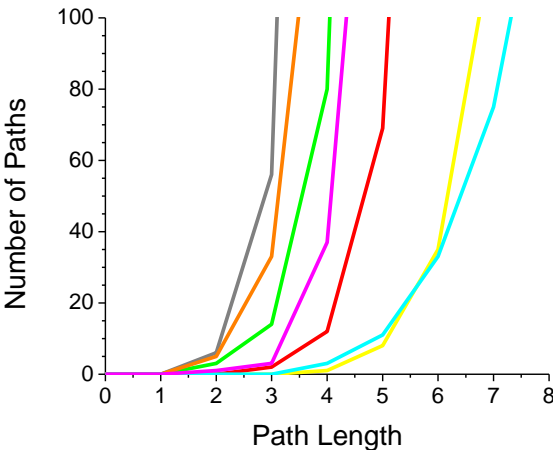

S1Ci

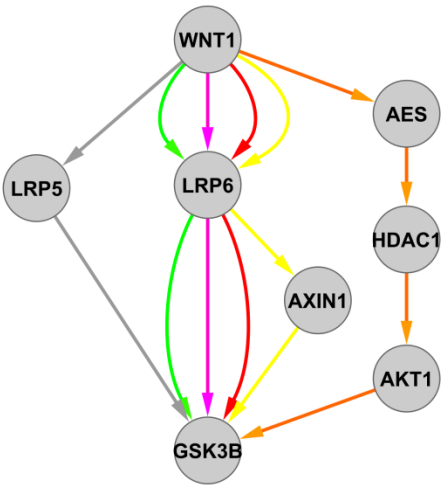

S1Cii

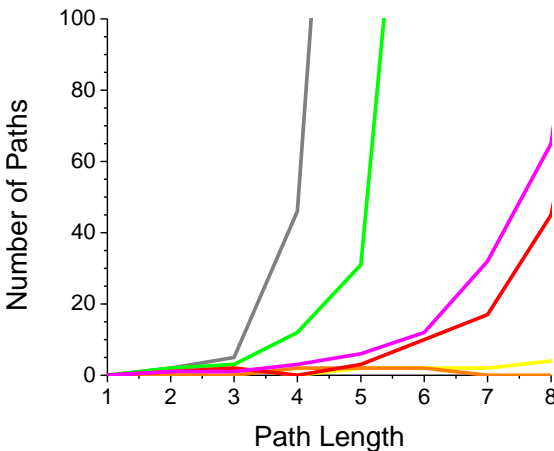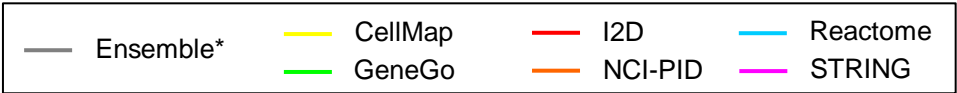

Supplement: Additional file 1: Figure S1 — Ensemble representation of TGFB, TNF, and WNT signalling networks. Alternate signal transmission routes connecting the extracellular ligands TGFB1 (A), TNF (B), and WNT (C) to their respective sentinel markers of pathway activity, SMAD4, NFKB1, and GSK3B. Shortest paths (i), and total number of paths as a function of path length (ii), color-coded by database source. [file 1752-0509-6-29-S1.pdf]
